# A Change of Heart: Human Cardiac Tissue Engineering as a Platform for Drug Development

**DOI:** 10.1007/s11886-022-01668-7

**Published:** 2022-03-05

**Authors:** Samantha B. Bremner, Karen S. Gaffney, Nathan J. Sniadecki, David L. Mack

**Affiliations:** 1 Department of Bioengineering, University of Washington, Seattle, WA USA; 2 Department of Mechanical Engineering, University of Washington, Seattle, WA USA; 3 Department of Laboratory Medicine and Pathology, University of Washington, Seattle, WA USA; 4 Center for Cardiovascular Biology, University of Washington, Seattle, WA USA; 5 Institute for Stem Cell and Regenerative Medicine, University of Washington, Seattle, WA USA; 6 Department of Rehabilitation Medicine, University of Washington, Seattle, WA USA

**Keywords:** Cardiac tissue engineering, Pluripotent stem cells, Drug screening, Cardiotoxicity

## Abstract

**Purpose of Review:**

Human cardiac tissue engineering holds great promise for early detection of drug-related cardiac toxicity and arrhythmogenicity during drug discovery and development. We describe shortcomings of the current drug development pathway, recent advances in the development of cardiac tissue constructs as drug testing platforms, and the challenges remaining in their widespread adoption.

**Recent Findings:**

Human pluripotent stem cell-derived cardiomyocytes (hPSC-CMs) have been used to develop a variety of constructs including cardiac spheroids, microtissues, strips, rings, and chambers. Several ambitious studies have used these constructs to test a significant number of drugs, and while most have shown proper negative inotropic and arrhythmogenic responses, few have been able to demonstrate positive inotropy, indicative of relative hPSC-CM immaturity.

**Summary:**

Several engineered human cardiac tissue platforms have demonstrated native cardiac physiology and proper drug responses. Future studies addressing hPSC-CM immaturity and inclusion of patient-specific cell lines will further advance the utility of such models for in vitro drug development.

## Introduction

The current drug development pathway is exorbitantly expensive and time-consuming. It is estimated that between 2009 and 2018, the median cost to bring a new drug to market was $985.3 million, including capitalized research and development investment costs [1]. This figure includes the cost of failed trials, as it has been reported that the approval rate for drugs entering phase I trials is only 13.8% [2]. Even having reached later phases of clinical trials, it is unfortunately common for drugs to fail due to lack of efficacy or unanticipated adverse effects, regardless of the promise shown in preclinical studies [3]. In a large part, this is due to limitations of animal models and in vitro preclinical models that limit our ability to accurately predict drug performance in human patients [4, 5]. Additionally, many pharmaceutical companies opt to embrace a fail early, fail fast strategy where the vast majority of drug candidates are eliminated early in the process to avoid costly late-stage failures, thus possibly missing potentially life-saving drugs [6]. In light of this, it is necessary that we re-evaluate the preclinical drug discovery and testing paradigm to make drug development more efficient and cost effective.

The shortcomings of the canonical drug development process are perhaps most evident when considering cardiac effects, as side effects such as cardiac toxicity and arrhythmogenicity are the most common reasons for late-stage drug failure or drug withdrawal [7, 8]. To address this, regulatory agencies require specific cardiotoxicity screening; however, the methods employed lack the relevance to the human cardiovascular system needed to accurately predict cardiac effects. The more simple in vitro models rely on non-cardiac cell lines that express a recombinant human *ether-á-go-go* related gene (hERG) potassium channel, as this channel plays a major role in cardiac repolarization and hERG-blocking compounds frequently cause ventricular arrhythmias [9, 10]. While useful in some cases, these simple, in vitro models are unable to model the interactions of multiple ion channels and the compensatory mechanisms present in the native myocardium. Increasing in complexity, rodent models are frequently used due to their relative low costs and short experimental timelines. However, these models often produce irrelevant results due to distinct physiological differences between human and rodent cardiovascular biology [5, 11]. While larger animal models can yield more relevant results, the costs and resources required for colony maintenance and ethical concerns are often prohibitive [12]. Thus, there exists a need to develop improved human in vitro cardiac models for drug screening and development.

The human heart is the ideal platform for drug testing; however, the limited availability and inadequate ex vivo viability of primary samples prevents their widespread use. Consequently, the development of human pluripotent stem cells (hPSCs, either embryonic stem cells, hESCs, or induced pluripotent stem cells, hiPSCs) and hPSC-derived cardiomyocytes (hPSC-CMs) holds great promise for advancing drug development platforms [13–16]. To date, hPSC-CMs have been shown to express key cardiomyocyte structural and signaling elements and faithfully recapitulate human cardiac biology, leading to their widespread use in studies demonstrating typical responses to drug compounds. However, hPSC-CMs are limited in their relative immaturity as compared to adult cardiomyocytes, as hPSC-CMs have been shown to more closely resemble fetal cardiomyocytes with regard to transcriptional activity, ultrastructure, and function, limiting their utility as a preclinical drug screening model [17–19]. Thus, several approaches have been employed to improve hPSC-CM maturity, including increased time in culture, topographical cues, biochemical stimuli, and the development of three-dimensional engineered models [20].

Human engineered cardiac tissues can be broadly defined as multicellular aggregates made from hPSC-CMs, often accompanied by other cell types, with or without the presence of extracellular matrix protein scaffolds. Such engineered constructs more closely mimic the native myocardium by recapitulating key cell–cell and cell–matrix biology that has been shown to further advance hPSC-CM maturation and facilitate key measures of cardiac function such as force production and voltage propagation [21–25]. These tissue constructs come in many shapes and sizes ranging from scaffold-free spheroids amenable to high-throughput screening to larger engineered chambers capable of generating pressure–volume loops. In recognition of the promise of these cardiac constructs to improve our ability to model cardiomyopathy and drug responses in vitro, research groups have developed various platforms, and many have moved toward commercialization. This review describes the newest advances made in the development of engineered cardiac constructs as valid platforms for preclinical drug screening and the remaining challenges preventing widespread adoption of these platforms.

## Engineered Cardiac Platforms for Drug Screening

**Fig. 1:**
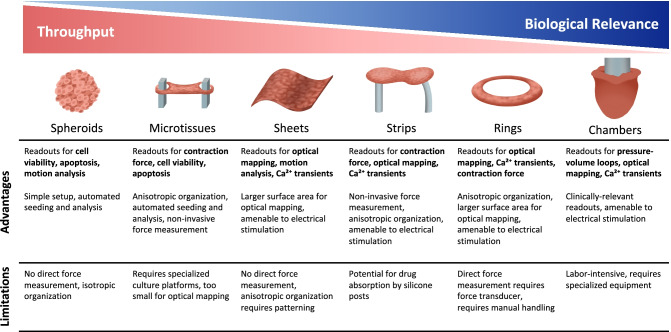
Different engineered cardiac tissue platforms organized by relative throughput and biological relevance with a description of their advantages and limitations

As a category, engineered cardiac tissues have come to include any cell culture platform facilitating multicellular, three-dimensional culture of synchronously contracting hPSC-CMs. As such, these platforms take many forms that vary widely in geometry and scale, the inclusion of non-myocytes, and the presence of scaffold proteins. Broadly, those on the smaller scale such as spheroids and microtissues more easily facilitate high-throughput cardiotoxicity screening while retaining some aspects of cardiac function, whereas larger platforms including cardiac sheets, strips, rings, and chambers are more suited to lower-throughput assessment of drug effect on cardiac function, as they more closely resemble native cardiac tissue and enable measurement of voltage propagation and force generation (Fig. 1). In the following sections, we have categorized these platforms broadly by geometry as a means to discuss their advantages and limitations as platforms for drug testing and progress made toward drug screening applications. Details describing the various platforms and findings from recent drug screening studies are summarized in Table 1.

- Spheroids
  At the smallest end of the scale are cardiac spheroids, also often referred to as cardiac organoids. Cardiac spheroids are small hPSC-CM aggregates that are formed by hanging-drop [26] or self-assembly on low-attachment substrates [27]. These platforms are generally scaffold-free, which allows for a dense network of cell–cell connections and removes any concern of drug absorption by scaffolding hydrogels or silicone support structures often used for larger platforms [28]. Spheroid systems often include non-myocyte cell types such as endothelial, fibroblast, and mesenchymal cells, which has repeatedly been shown to enhance spheroid function [29–31]. Perhaps most attractively, the small scale of cardiac spheroids requires significantly lower resources in terms of cell number and culture space and is most easily amenable to automated generation and high-throughput analysis.
  Cardiac spheroids have been used extensively to develop drug testing platforms. In an earlier study, it was shown that cardiac spheroids generated from hiPSC-CMs were similarly able to model doxorubicin-mediated cardiotoxicity as compared to spheroids made from primary human cardiomyocytes [32]. Demonstrating their high-throughput capabilities, a study used cardiac spheroids to screen a panel of 29 compounds approved by regulatory agencies with or without known structural cardiotoxicity [33•]. It was demonstrated that this platform was able to detect changes in cellular viability, endoplasmic reticulum integrity, and mitochondrial membrane potential. Beyond structural effects, cardiac spheroids have been used to stratify pro-arrhythmic toxicity of hERG channel blockers and environmental toxins [34].
  Using cardiac spheroids, it is also possible to model disease states. To model cardiac fibrosis, spheroids generated from hESC-CMs and hESC-derived mesenchymal stem cells were treated with transforming growth factor beta (TGF-β) [31]. It was shown that TGF-β triggered fibrotic features in the cardiac spheroids and that this response was worsened with the administration of known cardiotoxins. In a separate study, cardiac spheroids were used to model myocardial infarction by culturing in hypoxic conditions and treating with noradrenaline [35]. It was shown that hypoxic conditions worsened doxorubicin-mediated cardiotoxicity, while an antifibrotic compound could reduce ischemic spheroid stiffness and asynchronicity.
  While the use of cardiac spheroids for drug screening is advantageous given their relative accessibility and high-throughput nature, these culture systems do not promote uniaxially aligned contractile machinery, and functional outputs are often limited to cell viability with some insight into contractility and arrhythmogenicity by measurement of spheroid deflection. However, it is possible for spheroids to serve as building blocks for higher order tissues, as was demonstrated with bioprinting of spheroids into larger cardiac rings [36]. Ultimately, higher-throughput spheroid systems may be best suited for earlier stages of drug compound testing.
- Microtissues
  To increase functional readout capacity while retaining the high-throughput benefits of spheroid culture systems, many have developed what we here call microtissues, where a similarly small number of cells, sometimes in a hydrogel scaffold, are self-assembled onto manufactured posts, such that they form geometries similar to the cardiac strips and rings presented in the following sections [37–41]. It was demonstrated that microtissues outperform age-matched two-dimensional hiPSC-CMs in terms of predictive accuracy in drug response [38]. Building on the high-throughput advantages of spheroid culture systems, these microtissue platforms provide uniaxial mechanical cues, generating improved cellular alignment and facilitate more rigorous measurement of contractile function by tracking the deflection of cantilever posts.
  Possibly the most useful application of microtissues or other smaller cardiac platforms is as an intermediate screen between high-throughput two-dimensional in vitro experiments and animal studies. This paradigm was demonstrated in a study screening for pro-proliferative compounds using 96-well microtissues, where an initial pool of approximately 5,000 compounds were screened in two-dimensional hiPSC-CMs for their ability to induce cellular proliferation [42•]. Of this initial pool, 105 compounds were identified and screened further for pro-proliferative effects using microtissues, which also allowed for the elimination of compounds causing negative functional effects. A smaller pool of the leading compounds were then further evaluated in microtissues that were further matured with fatty acid supplementation [40, 42•]. This study uniquely demonstrates a pathway by which to pursue drug development with varying hierarchies of in vitro hPSC-CM models.
- Sheets
  Cardiac sheets consist of one or multiple layers of hPSC-CMs and are particularly useful for detecting arrhythmogenicity. Using fluorescent voltage or calcium-sensitive dyes or genetically encoded sensors, it is possible to model arrhythmic risk of drug compounds by visualizing conduction speeds and re-entry waves as was done in two-dimensional cell sheets [43]. This platform has the additional benefit of microgrooves providing anisotropic cell patterning that more closely represents native myocardium. Other groups have developed methods of coating hPSC-CMs and other cell types with ECM and seeding them into cell sheets that are multiple layers thick [44, 45]. Using motion tracking, it was possible to measure the effects of several drug compounds on magnitude of contraction, contraction kinetics, and abnormal beat intervals [44]. However, shortcomings of cardiac sheets include the difficulty in obtaining direct measurement of force output and the need for additional interventions to facilitate sheet patterning and cellular alignment.
- Tissue Strips
  Cardiac strips are perhaps the most commonly thought of hEHT platform along with spheroids and are made from hPSC-CMs embedded in a hydrogel that is cast into a mold where it solidifies and subsequently compacts and begins beating spontaneously [46]. Cardiac strips are cast uniaxially between two elastomeric posts [47] or wires (Biowire) [48, 49]. This platform enables higher-throughput measurement of contractile forces via tracking the deflection of elastomeric supports. As such, these models are particularly suited to drug screening, where easily measured force production and kinetics can provide insights into the inotropic and arrhythmogenic effects of test compounds.
  To date, cardiac strips have been used to test a wide panel of compounds with and without known cardiac effects. A panel of eleven compounds was tested on cardiac strips, demonstrating that these tissues were able to faithfully reproduce positive and negative inotropic effects when compared to human atrial trabeculae, but the relative immaturity of the hiPSC-CMs was evident due to limited observed beta-adrenergic effects [50]. The benefits of three-dimensional culture were further demonstrated in another study where an impressive panel of 28 drugs was tested on cardiac strips as well as 2D hiPSC-CM monolayers, where it was demonstrated that the tissues yielded more accurate drug responses in terms of contractility and calcium transient response (85% accuracy for hiPSC-CM monolayers vs. 93% accuracy for tissue strips) [51]. Additionally, both of these studies demonstrate the potential for increased throughput with tissue strip platforms despite their larger size, as not only were multiple drugs tested, but at multiple doses, enabling the derivation of EC50 values.
  Chronic electrical stimulation of cardiac strips has been demonstrated to improve tissue maturity and promote positive force-frequency relationships, thus improving the accuracy of drug responses and the ability to model positive inotropy [52•]. Similar stimulation protocols have been used on the Biowire II platform, which was used to derive EC50 values and demonstrate canonical responses for several drug compounds [49]. This platform and others have been further developed to model specific atrial and ventricular responses to drugs in chamber-specific tissues [53, 54] and to explore anti-fibrotic drugs in angiotensin II-mediated non-genetic cardiomyopathy [55]. Despite their increased size and required resources as compared to spheroids or microtissues, strip hEHTs have been used to generate impressive datasets demonstrating relevant responses to many compounds at various doses in a single study. Paired with efforts to improve hEHT maturity, efforts to increase analysis throughput through optical [56, 57] or magnetic detection of post movement [58], will only further increase the utility of cardiac strip platforms.
- Tissue Rings
  Cardiac rings are similar to cardiac strips save for their shape, where cardiac rings are cast in circular molds before being transferred to isometric or elastomeric supports [59]. Given their larger surface area, cardiac rings are particularly suited to modeling voltage propagation as an indicator of arrhythmogenicity. Using genetically encoded voltage and calcium sensors, tissue rings have been shown to properly model drug-induced changes in contraction rate and conduction properties [60]. Additionally, by using patient-derived hiPSCs, authors were able to reproduce long QT syndrome and demonstrate drug-induced reentrant arrhythmias. This platform was further developed to model chamber-specific responses to a panel of drugs by using atrial or ventricular hiPSC-CMs [61]. While tissue ring platforms are useful for modeling drug-induced changes in conduction properties and arrhythmogenicity, it would appear that they may be less popular than other platforms of similar size. This may be due to the requirement for more individual tissue handling, as measurement of contractile forces often requires the use of a force transducer, which hampers throughput.
- Chambers
  At the opposite end of the spectrum from cardiac spheroids are cardiac chambers, which resemble a miniaturized ventricle [62, 63]. The geometry of this platform most closely resembles a native ventricle, and engineered cardiac chambers are the only platform capable of generating pressure, enabling measurement of clinically relevant outputs including ejection fraction, cardiac output, and pressure–volume loops. Cardiac chambers have been shown to surpass lower-order 2D and 3D hPSC-CM culture systems in transcriptional maturity [63]. Given their advanced maturation and attainable performance metrics, chamber constructs are an appealing platform for drug screening and characterization.
  One such platform (human ventricle-like cardiac organoid chamber, hvCOC) is generated by casting hESC-CMs in a hydrogel around a balloon catheter, which is removed after tissue compaction [63]. This initial study demonstrated altered pressure–volume loops and electrophysiological performance after treatment with six compounds. In a follow-up study, hvCOCs and human ventricular-like cardiac tissue strips (hvCTS) were treated with a panel of 25 cardioactive compounds, where it was demonstrated that hvCOCs displayed enhanced positive inotropy as compared to hvCTSs [64•]. A similar platform has been developed using pull-spun nanofibers that recapitulate the concentric, anisotropic orientation of native myocardium [62]. While it was possible to measure pressure–volume loops, this model failed to replicate a positive inotropic response with isoproterenol treatment, indicating relative immaturity.
  Compared to other cardiac tissue platforms, cardiac chambers are limited in terms of the increased resources and technical expertise required, ultimately resulting in a lower-throughput platform. However, with the addition of additional maturation techniques such as electrical stimulation and anisotropic cell sheet patterning [65], such models could effectively serve as in vitro replacements for Langendorff whole-heart preparations.

**Table 1:** Summary of drug screening studies in human engineered cardiac tissues

Construct type	Cell types	Cells per hEHT	Scaffold material	Compounds tested and dosage				Readouts	Ref.
Spheroid	hiPSC-CM HCAEC iPSC-CF	1.2 × 10^4^	Scaffold-free	Doxorubicin L-NIO	1–40 µM 100 µM			• Cell viability • Apoptosis	[32]
Spheroid	hiPSC-CM hCMEC hCF	5 × 10^2^	Scaffold-free	Acyclovir Amiodarone Amphotericin B Bortezomib Buspirone Cisapride Clozapine Cyclophosphamide Dasatinib Donepezil Doxorubicin Erlotinib Fluorouracil Gemfibrozil Idarubicin	0.3–100 µM 0.2–50 µM 0.2–50 µM 0.3–100 µM 0.3–100 µM 0.3–100 µM 0.3–100 µM 0.3–100 µM 0.3–100 µM 0–30 µM 0.2–50 µM 0–30 µM 0.3–100 µM 0.3–100 µM 0.3–100 µM	Imatinib Isoproterenol Ketoprofen Lapatinib Mebendazole Methapyrilene Minoxidil Mitoxantrone Naringenin Nifedipine Praziquantel Sorafenib Sunitinib Terfenadine	0.2–50 µM 0.3–100 µM 0.3–100 µM 0.3–100 µM 0.2–50 µM 0.3–100 µM 0.2–50 µM 0.3–100 µM 0.3–100 µM 0.3–100 µM 0.3–100 µM 0.3–100 µM 0.1–30 µM 0.3–100 µM	• Cellular viability • Mitochondrial membrane potential • Endoplasmic reticulum integrity	[33•]
Fibrotic spheroid	hESC-CM hESC-MSC	5 × 10^4^	Scaffold-free	Aldosterone Bisphenol A Metoprolol	10 µM 10 µM 10 µM			• Collagen deposition • Apoptosis • Mitochondrial morphology	[31]
Ischemic spheroid	hiPSC-CM NHCF HUVEC hADSC	4.3 × 10^4^	Scaffold-free	Doxycycline JQ1	0.1–50 µM 10 nM			• Contraction amplitude • Apoptosis	[35]
Spheroid	hiPSC-CM hCF	1.2–2.4 × 10^4^	Scaffold-free	4-Aminopyridine BayK8644 BPA	50 mM 300 nM 1–1000 nM	E4031 Isoproterenol Ranolazine	2 µM 100 nM 2–100 µM	• Optical mapping of membrane potential	[34]
3D printed spheroid array	hiPSC-CM HUVEC NHDF	3.5 × 10^4^ per spheroid 500 spheroids per tissue	Scaffold-free	Blebbistatin Doxorubicin Isoproterenol Propranolol	500 nM 10 µM 1 µM 5 µM			• Contraction amplitude and kinetics • Cell viability	[36]
Microtissue strip	hiPSC-CM	0.9–1.8 × 10^6^	Scaffold-free	Ampicillin E4031 Erythromycin Metformin Rosiglitazone	0.01 µM–1 mM 0.001–10 µM 0.01–500 µM 0.01–1 mM 0.001–200 µM	Tamoxifen Troglitazone Trovafloxacin Vandetanib	0.001–200 µM 0.01–200 µM 0.01–200 µM 0.001–100 µM	• Contraction amplitude • Beating rate • Cell viability	[38]
Microtissue strip	hiPSC–CM hMSC	5 × 10^2^	Collagen I	Sunitinib	1–10 µM			• Apoptosis • Beating rate	[41]
Microtissue strip	hESC-CM	5 × 10^4^	Collagen I Matrigel	105 compounds	0.1–10 µM			• Contraction amplitude • Proliferation	[42•]
Microtissue ring	hESC-CM hCF	7.5 × 10^4^	Collagen I	Blebbistatin Isoproterenol	1 nM–10 µM 10 nM–1 µM	Nifedipine	1 nM–10 µM	• Contraction amplitude	[37]
Anisotropic cell sheet	hESC-CM	2.5 × 10^5^ cm^−1^	Scaffold-free	Aspirin Cisapride Flecainide	3–30 µM 30–300 nM 0.3–3 µM	Procainamide Terfenadine Tocainide	30–300 µM 30–300 nM 10–100 µM	• Action potential kinetics	[43]
Layered cell sheet	hiPSC-CM NHCF HMVEC	1 × 10^6^	Fibronectin Gelatin	E-4031 Isoproterenol	1–100 nM 1–1000 nM			• Beating rate • Contraction kinetics	[45]
Layered cell sheet	hiPSC-CM NHCF HMVEC	1.1 × 10^6^	Fibronectin Gelatin	Chromanol 293B Dobutamine E-4031 Flecainide Glibenclamide	1–30 nM 3–100 µM 3–30 nM 30–1000 nM 1–30 µM	Isoproterenol Milrinone Ouabain Pimobendan Verapamil	1–1000 nM 3–100 µM 3–30 nM 0.1–3 µM 3–100 nN	• Beating rate • Contraction kinetics • Contraction amplitude	[44]
Tissue strip	hiPSC-CM HS27a	5.5 × 10^5^	Fibrin	Isoproterenol Verapamil	0.05–50 µM 0.5–50 µM			• Beat Rate • Contraction force	[58]
Tissue strip	hiPSC-CM	1 × 10^6^	Matrigel Fibrin	Aspirin Citalopram Digoxin Isoproterenol Formoterol Lidocaine	0.1–30 µM 0.1–30 µM 0.1–30 µM 3–300 nM 0.01–3 µM 0.1–30 µM	Milrinone Nifedipine Rolipram Ryanodine Tacrolimus	0.1–30 µM 0.1–30 µM 0.1–30 µM 0.1–30 µM 0.1–30 µM	• Contraction force • Contraction kinetics	[50]
Tissue strip	hPSC-CM	1 × 10^6^	Matrigel Fibrin	BayK-8644 Digoxin EMD-57033 Isoproterenol	3–300 nM 0.01–1 µM 0.1–10 µM 0.3–100 nM	Nifedipine Ryanodine Thapsigargin	3–1000 nM 1–30 µM 3–300 µM	• Contraction force • Contraction kinetics	[78]
Atrial tissue strip	hiPSC-CM	1 × 10^6^	Fibrin	4-AP CCh	50 µM 10 µM			• Action potential amplitude and kinetics	[54]
Tissue strip	hiPSC-CM NHDF	2 × 10^6^	Fibrin	Isoproterenol	0.01 nM–1 mM			• Beat rate • Contraction force	[52•]
Tissue strip (Biowire)	hiPSC-CM NHCF	1.1 × 10^5^	Fibrin Collagen Matrigel	Digoxin Dobutamine Endothelin-1 FPL64176 Isoproterenol Levosimendan	0.1 nM–100 µM 0.003 nM–3 µM 0.0004–40 nM 0.1 nM–30 µM 1 pM–10 µM 0.2 nM–20 µM	Mavacamten Milrinone Nifedipine Omecamtiv PACAP27 Thapsigargin	0.1 nM–100 µM 1 nM–300 µM 0.01 nM–10 µM 1 nM–10 µM 0.001–300 nM 0.1 nM–30 µM	• Contraction force	[49]
Atrial and ventricular tissue strip (Biowire)	hESC-CM NHCF	1.1 × 10^5^	Collagen Matrigel	4-AP Carbachol Diltiazem Dofetilide E4031 Isoproterenol	25–50 µM 1 µM 10–20 µM 10–1000 nM 0.01 nM 0.1–10 nM	Lidocaine Milrinone Nifedipine Serotonin Thapsigargin Verapamil	10–20 µM 0.08–20 µM 0.01–10 µM 0.01–1 µM 5–50 µM 0.1–10 µM	• Action potential amplitude and kinetics • Contraction force • Ca^2+^ transients	[53]
Tissue strip (Biowire)	hiPSC-CM NHCF	1.1 × 10^5^	Fibrin Matrigel	Angiotensin II Losartan	200 nM 10–50 µM	Relaxin Saracatinib	0.1–0.5 µg/mL 1–10 µM	• Contraction force and kinetics • Beat rate • Ca^2+^ transients • Cell viability	[55]
Tissue strip	hiPSC-CM	1 × 10^6^	Fibrin Matrigel	Aspirin Atenolol Captopril Citalopram Clonidine Dobutamine Doxorubicin Enalaprilat Epinephrine Flecainide Forskolin Glibenclamide Itraconazole Ivabradine	0.01–1 mM 0.1–10 µM 1–100 µM 1–100 µM 0.01–1 µM 0.1–10 µM 0.1–10 µM 1–100 µM 0.01–1 µM 0.1–10 µM 0.1–10 µM 0.1–10 µM 0.1–10 µM 0.1–10 µM	Levosimendan Milrinone Omecamtiv Paracetamol Phentolamine Pimobendan Pravastatin Sildenafil Sorafenib Sunitinib Terbutaline Tolbutamide Verapamil Zimelidine	0.01–1 µM 1–100 µM 0.01–1 µM 10–1000 µM 1–100 µM 1–100 µM 1–100 µM 0.3–30 µM 0.1–10 µM 0.1–10 µM 0.1–10 µM 1–100 µM 0.01–1 µM 1–100 µM	• Contraction amplitude and kinetics	[51]
Tissue ring	hiPSC-CM	2 × 10^6^	pcECM Chitosan	ATX-II Carbamylcholine Carbenoxolone Dofetilide E-4031	30 nM 1 µM 50 µM 25 nM 0.1–1 µM	Isoproterenol Lidocaine Ouabain Quinidine	1 µM 100 µM 1 mM 0.1–30 µM	• Ca^2+^ transients • Action potential amplitude and kinetics • Contraction force and kinetics	[60]
Atrial and ventricular tissue ring	hESC-CM	2 × 10^6^	Collagen	Carbamylcholine Flecainide Isoproterenol	2–10 µM 10 µM 10 µM	Lidocaine Nifedipine Vernakalant	100 µM 0.1 µM 30 µM	• Action potential duration • Beat rate • Contraction force	[61]
Chamber	hiPSC-CM	3 × 10^6^	PCL/gelatin nanofibers	Isoproterenol	0.1 nM–0.1 mM			• Pressure–volume loops • Beat rate	[62]
Chamber (hvCOC)	hESC-CM NHDF	1 × 10^7^	Collagen I Matrigel	Digoxin Disopyramide Flecainide	0.1 µM 1 µM 0.01–10 µM	Isoproterenol Nifedipine Verapamil	0.01–10 µM 1 µM 0.01–1 µM	• Pressure–volume loops • Action potential amplitude and kinetics	[63]
Chamber (hvCOC)	hESC-CM NHDF	1 × 10^7^	Collagen I Matrigel	Isoproterenol Levosimendan	0.1–10 µM 0.001–1 µM	Milrinone	0.01–1 µM	• Pressure–volume loops	[64•]
Tissue strip (hvCTS)	hESC-CM NHDF	1 × 10^6^	Collagen I Matrigel	Amitriptyline Aspirin Bepridil Caffeine Digoxin Disopyramide Dobutamine Dopamine Flecainide Glibenclamide Isoproterenol Levosimendan Lidocaine	10–100 µM 0.03–30 µM 30–300 µM 0.1–100 µM 0.1–30 µM 0.1–100 µM 0.03–100 µM 1–100 µM 0.1–100 µM 0.1–30 µM 0.001–3 µM 0.1–30 µM 0.03–1 mM	Lisinopril Mibefradil Milrinone Nifedipine Norepinephrine Pravastatin Procainamide Quinidine Ramipril Tocainide Tolbutamide Verapamil	0.1–30 µM 0.1–10 µM 0.03–100 µM 0.03–10 µM 1–10 µM 0.03–30 µM 0.1–100 µM 3–100 µM 0.1–30 µM 0.1–100 µM 0.1–3 µM 0.03–3 µM	• Contraction force	[64•]

*hADSC* human adipose-derived stem cells (Lonza), *HCAEC* human coronary artery endothelial cells (Cell Applications), *hCF* human cardiac fibroblasts (PromoCell), *hCMEC* human cardiac microvascular endothelial cells (PromoCell), *hESC-CMS* human embryonic stem cell derived mesenchymal stem cell, *hiPSC-CF* hiPSC-derived cardiac fibroblasts (Axiogenesis), *hMSC* human mesenchymal stem cells (Lonza), *HMVEC* human cardiac microvascular endothelial cells (Lonza), *HS27a* human bone marrow stromal cells (ATCC), *HUVEC* human umbilical vein endothelial cells (Lonza), *hvCOC* human ventricular cardiac organoid chamber, *hvCTS* human ventricular cardiac tissue strip, *NHCF* normal human ventricular cardiac fibroblasts (Lonza), *NHDF* normal human dermal fibroblasts (Lonza), *pcECM* porcine cardiac ECM, PCL polycaprolactone

## Challenges Remaining

Despite wide-spread excitement surrounding engineered cardiac platforms for drug development and several commercialization efforts underway, several hurdles remain. Compared to adult myocardium, or even neonatal myocardium, engineered cardiac constructs present a very immature phenotype, potentially limiting their physiological relevance. In early stages after differentiation with no intervention, hPSC-CMs display only fetal transcriptomes [66], ion channel expression [67], metabolic function [68], and contractility [69]. While three-dimensional culture has been shown to promote advanced hPSC-CM maturation [21–25], it is evidently insufficient to routinely produce a robust cardiac phenotype that includes positive force-frequency responses and positive inotropic responses, thus potentially limiting their use as drug screening platforms. Several methods shown to advance hPSC-CM maturity in two-dimensional culture could potentially have the same effect in tissues, including fatty acid [70] or thyroid hormone supplementation [71] or microRNA treatment [72, 73]. Advanced engineering approaches have been employed to further increase the maturity achieved in cardiac tissues, including electrical stimulation [52•], increasing afterload [74], and the addition of preload or passive stretch [75, 76], which, if successful, will greatly improve the predictive capacity of these engineered models.

A significant difficulty that has become apparent when working with engineered tissues is controlling variability and demonstrating reproducibility. This variability arises, in part, from hPSC-CM batch-to-batch variability and the different protocols used by different institutions for hPSC-CM differentiation and tissue generation, both of which are inherently human processes where results may vary simply by the hands performing the experiment. To remove human sources of variability, many are turning toward automation of tissue generation and analysis, which will also increase platform throughput [37, 57, 77]. Additional variation arises from the different genetic backgrounds of the various hPSC lines used. Highlighting this challenge, a study compared the performance of ten different hPSC lines in cardiac tissue strips [78]. It was found that spontaneous and electrically paced tissue contractile performance and kinetics varied widely between the different lines, emphasizing the need for isogenic controls in disease modeling and advocating for the use of multiple hPSC lines during platform validation. Interestingly, it was found that despite the variability in baseline performance, the different hPSC lines behaved more consistently with regard to drug response, although with varying EC50 values [78]. To address this concern, it is likely that robust cardiac tissue platforms will continue to rely on multiple biological replicates and turn toward automation and the use of multiple genetic backgrounds.

Conversely, this variability can be seen as a facet of hPSC and tissue engineering that has not yet been fully taken advantage of. There are numerous studies describing the development of hPSC lines harboring cardiomyopathy-associated mutations that could be further used to screen disease-specific drug candidates in engineered cardiac constructs [79]. Additionally, such engineered tissue models can be used to examine biological sex-related differences in cardiac biology and disease, as these differences are known, but often overlooked in in vitro disease modeling and preclinical screening [80]. By including multiple cell lines from different genetic backgrounds and with different disease-causing genetic variants, we can begin to approximate not only personalized and patient-specific medicine, but also population-wide responses to different pharmacological agents in the dish.

A significant limitation to translatability is that most platforms lack the biological complexity needed to fully reproduce native myocardium, necessitating the continued reliance on animal models for preclinical testing. At their simplest, cardiac constructs contain only hPSC-CMs, while others have included additional cell types including fibroblasts, endothelial cells, and mesenchymal cells, which have been shown to improve tissue quality and maturity. A biological element often missing from engineered cardiac constructs is vasculature, a key component needed to accurately model drug delivery. Strategies to vascularize constructs consist of co-culturing with endothelial cells and addition of angiogenic factors, three-dimensional bioprinting, or microfluidic systems [81, 82]. The development of in vitro models that better recapitulate the complexity of drug delivery and toxicity will also require the inclusion of additional organ systems involved in drug metabolism and clearance, e.g., hepatic and renal systems. To achieve this, several groups are developing complex, modular organ-on-a-chip systems [83, 84]. If successful, such vascularized multi-organ systems would be the pinnacle of in vitro drug testing platforms.

Lastly, it is worth noting that the vast majority of studies described in this review evaluated only compounds with known effects in human patients. As such, further studies are needed to demonstrate the true predictive capabilities of engineered cardiac tissues for clinical trial success. However, some pioneering studies have used engineered constructs to explore novel antifibrotic agents [55], perform screening experiments to identify pro-proliferative compounds for heart regeneration [42•], evaluate a novel myotrope [85], and even evaluate the effect of COVID-19 treatments on cardiac function [86]. Thus, it is evident that the stage is now set to explore the potential effects of novel therapeutics in engineered cardiac platforms.

## Conclusion

In order to increase the efficiency with which new drugs are discovered and brought to clinical trials, it is necessary to improve the human in vitro models used, to increase their biological relevance and enable the field to move away from a reliance on animal models as the gold standard. To address concerns of cardiac toxicity, great efforts have been put toward the development of engineered cardiac tissues from hPSC-CMs. These platforms range in shape and scale from cardiac spheroids and microtissues, to sheets, strips, and rings, to chambers emulating an entire ventricle, all of which present their own advantages and limitations. This review has highlighted recent advances made in the development of cardiac tissue engineering for drug screening platforms. While lacking functional maturity and structural complexity in some regards, these constructs hold merit as drug screening platforms with powerful predictive capabilities that, as they stand, can provide value to early stages of the drug screening pipeline. With continued advancements in tissue maturity, automation, and throughput, it is our prediction that cardiac tissue engineering will continue to gain favor in the pharmaceutical industry.
